# Supplementary material for: The causal relationship between the human gut microbiota and pyogenic arthritis: a Mendelian randomization study
Source: Front Cell Infect Microbiol. 2024 Nov 26;14:1452480. doi: 10.3389/fcimb.2024.1452480 (PMC11629706; doi:10.3389/fcimb.2024.1452480)
Supplement: Supplementary file 1 [file DataSheet1.zip › mendelian test/ebi-a-GCST90027769.csv_forest.pdf]

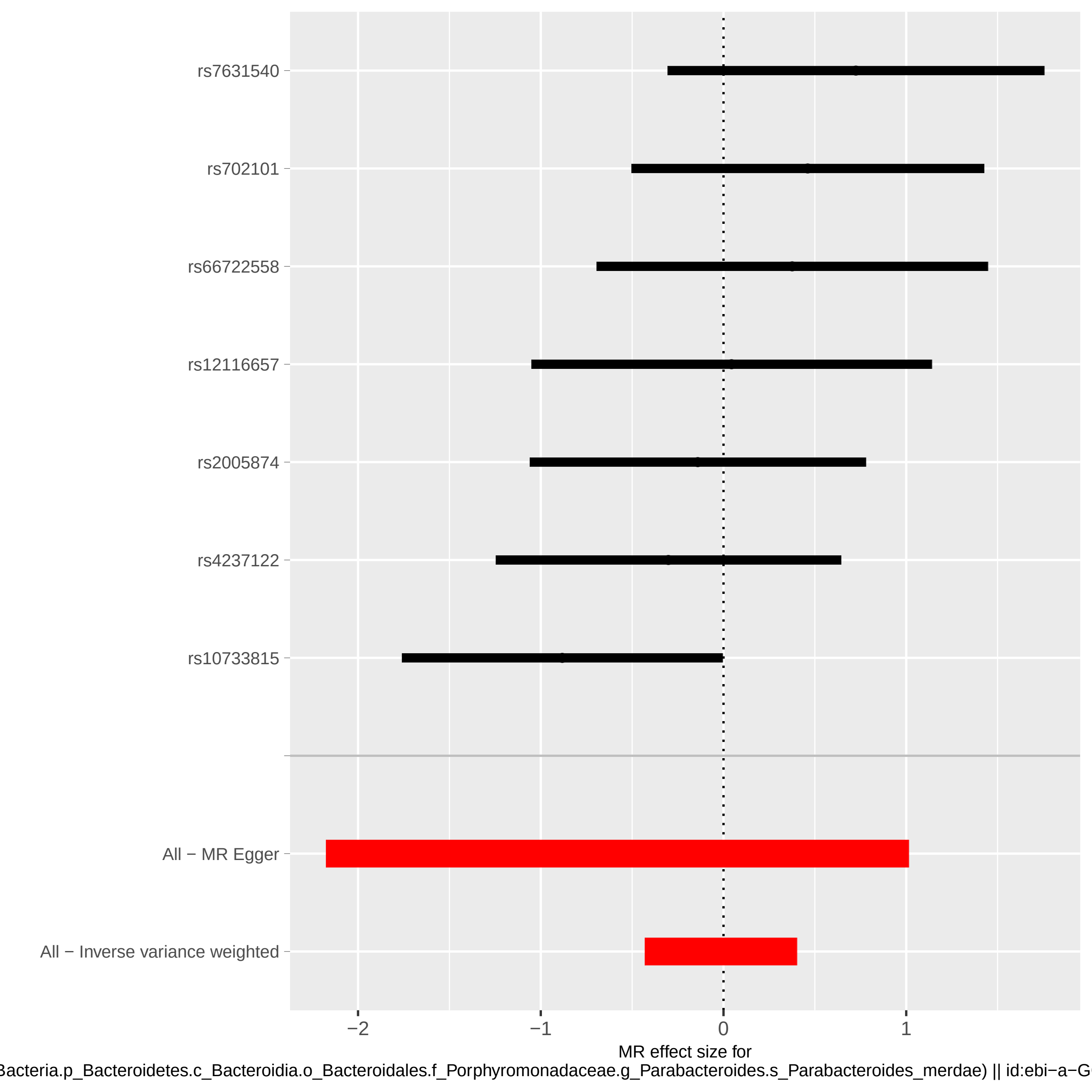
rs7631540
rs702101
rs66722558
rs12116657
rs2005874
rs4237122
rs10733815
All − MR Egger
All − Inverse variance weighted
−2
−1
0
1
MR effect size for
Bacteria.p_Bacteroidetes.c_Bacteroidia.o_Bacteroidales.f_Porphyromonadaceae.g_Parabacteroides.s_Parabacteroides_merdae) || id:ebi−a−G
